# Long-Term Swallowing Outcome and Dysphagia in Advanced Staged Head and Neck Squamous Cell Carcinomas after Radiotherapy

**DOI:** 10.3390/jcm11102688

**Published:** 2022-05-10

**Authors:** Erdem Yildiz, Stefan Grasl, Doris-Maria Denk-Linnert, Gabriela Altorjai, Harald Herrmann, Matthaeus C. Grasl, Boban M. Erovic, Stefan Janik

**Affiliations:** 1 Department of Otorhinolaryngology, Head and Neck Surgery, Medical University of Vienna, 1090 Vienna, Austria; erdem.yildiz@meduniwien.ac.at (E.Y.); stefan.grasl@meduniwien.ac.at (S.G.); matthaeus.grasl@meduniwien.ac.at (M.C.G.); 2 Department of Otorhinolaryngology, Division of Phoniatrics and Speech Language Therapy, Medical University of Vienna, 1090 Vienna, Austria; doris-maria.denk-linnert@meduniwien.ac.at; 3 Department of Radiation Oncology, Medical University of Vienna, 1090 Vienna, Austria; gabriela.altorjai@meduniwien.ac.at (G.A.); harald.herrmann@meduniwien.ac.at (H.H.); 4 Institute of Head and Neck Diseases, Evangelical Hospital, 1180 Vienna, Austria; boban.erovic@meduniwien.ac.at

**Keywords:** squamous cell carcinoma, head and neck cancer, swallowing disorder, dysphagia, adjuvant therapy, radiotherapy

## Abstract

Objective: To evaluate the impact of radiotherapy (RT) on dysphagia and long-term swallowing outcome in patients with stage III and IV head and neck squamous cell carcinomas (HNSCCs). Material and Methods: Between 2005 and 2008, 189 patients with HNSCCs underwent primary or adjuvant RT in a curative setting. Long-term swallowing outcome was evaluated in 50 patients. Among them, 26 were further eligible for prospective analysis of long-term swallowing and dysphagia outcome. Medical charts were retrospectively reviewed regarding pre- and post-treatment dysphagia (3 months after last irradiation setting) as well as persisting long-term dysphagia (2019–2021). Results: Pre-treatment dysphagia was observed in 24 (48%) of 50 patients, particularly in oropharyngeal or hypopharyngeal stage III–IV tumors (OR 9.3; *p* = 0.003). Conversely, 46 patients (92%) complained about post-treatment dysphagic symptoms, which were more commonly seen in patients with positive neck nodes (OR 10.5; *p* = 0.037). The post-treatment dysphagia rate dropped from 92% to 24% (*p* < 0.001) during surveillance, which was significantly linked to xerostomia (OR 5.77; *p* = 0.019), dysgeusia (OR 9.9; *p* = 0.036) and free flap reconstruction (OR 6.1; *p* = 0.022). Conclusion: Pretreatment dysphagia is common in advanced stage HNSCCs and almost all patients complain about dysphagia at the end of RT. Importantly, applied RT protocols did not affect long-term dysphagia, which improves significantly in the majority of patients over time. Meeting Information: Preliminary results have been presented at the 65th Annual Meeting of the Austrian Society of Otorhinolaryngology, 22–26 September 2021, Austria.

## 1. Introduction

Dysphagia and swallowing disorders are typically experienced by patients with head and neck cancer (HNC). These may result either from tumor extension and invasion or as treatment-related sequelae. It is well known that swallowing malfunctions are recognized as a significant burden and major limiting factor of patients’ quality of life (QoL) [1,2]. Adjuvant therapy, especially chemoradiotherapy, significantly impacts the overall survival of HNC patients [3]. As the impact not only applies to the overall outcome but also to the patients’ QoL [4], adjuvant therapy opens up an essential basis for treatment choice. Hence, QoL is significantly affected in HNC patients if swallowing is affected.

Dysphagia, in turn, describes any difficulty or discomfort regarding swallowing and represents, therefore, first and foremost, a symptom of the disease. Clinical manifestations range from the disability of oral nutrition and G-tube dependence to mostly unaffected swallowing. To overcome this issue hampering serious comparisons, the penetration-aspiration scale (PAS) was established to classify the severity of dysphagia according to an 8-point Likert scale [1,2,3,4,5,6,7,8]. The PAS is nowadays widely used for the interpretation of videofluoroscopy (VFS) examinations and for fiberendoscopic (flexible) examinations of swallowing (FEES), although PAS may also differ between VFS and FEES studies [6,7].

Patients with advanced-stage HNC (stage III and IV) often experience severe long-term sequelae after multimodal or invasive therapies comprising radiotherapy (RT), either in combination with chemotherapy or adjuvant after primary surgery. Numerous side-effects, like mucositis, xerostomia, and soor, are particularly associated with chemoradiation resulting in dysphagia as well as with RT-induced tissue changes that seem to persist [9,10,11].

As RT is assumed to decrease QoL by causing swallowing malfunctions significantly, we aimed to assess the impact of different RT protocols on the occurrence of dysphagia and whether dysphagia persists or resolves over time. The primary aim of our study was to investigate the effects of different treatment modalities (primary RT vs. surgery and PORT ± CRT) on short- and long-term dysphagia in advanced staged HNC patients. Secondary, we aimed to identify potential factors that may add to the risk of dysphagia occurrence and persistence.

## 2. Material and Methods

### 2.1. Study Cohort

**Figure 1:**
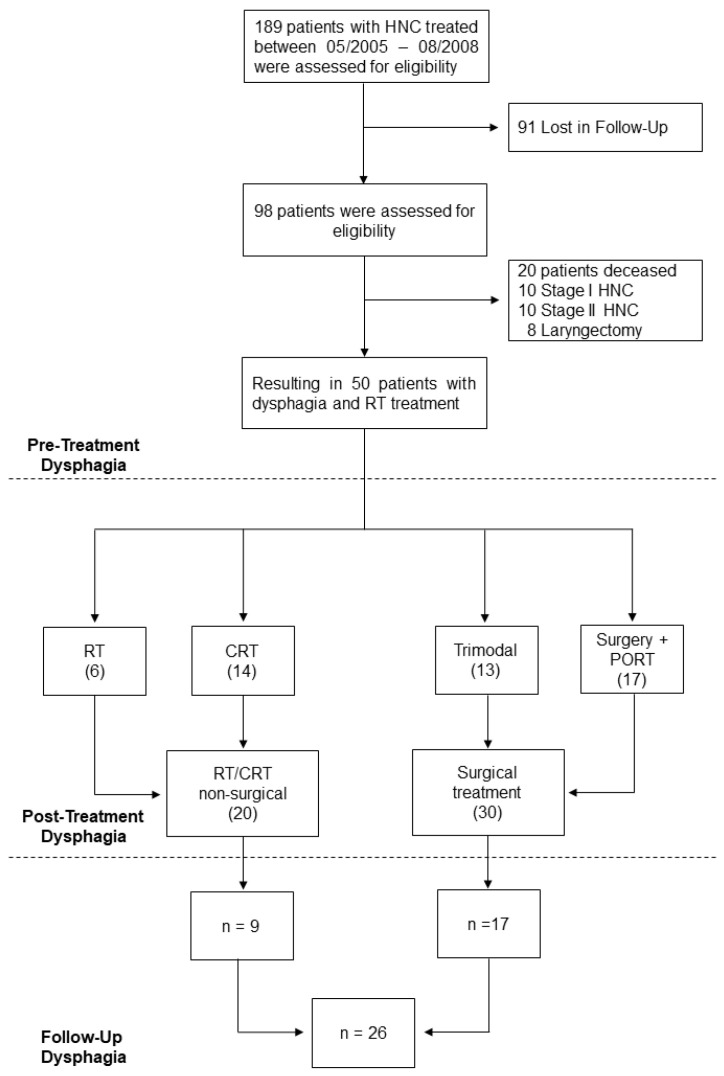
Flow-Chart. Time period in figure refers to May 2005–August 2008. The number in brackets refer to quantity of patients included in each group. Abbreviations: CRT, chemoradiotherapy; HNC, Head and Neck Cancer; PORT, post-operative radiotherapy; RT, radiotherapy.

189 HNSCC patients underwent RT between May 2005 and August 2008 at the Vienna General Hospital, Austria [12,13,14] and were therefore evaluated for eligibility. Those with missing data, the occurrence of recurrence, or insufficient follow-up time of fewer than 60 months were initially excluded (*n* = 91). Secondly, we excluded stage I (*n* = 10) and stage II (*n* = 10) tumors as we were interested in advanced-stage diseases. Patients who deceased (*n* = 20) and those who underwent laryngectomy (*n* = 8) were further excluded. Finally, 50 patients with stage III and IV HNSCCs were included (Figure 1).

### 2.2. Clinical Data

Clinical and sociodemographic data were retrospectively collected from electronic patient records within the treatment period and from outpatient reports during regular follow-up examinations (Table 1). We systemically screened appropriate patients’ records with regards to dysphagia or swallowing malfunctions. All patients were weekly interviewed regarding any nutrition-related problems, like problems with swallowing solid food/liquids, weight loss, reduced appetite, or coughing during oral intake. RT-induced side effects, namely soor, dysgeusia, erythema, xerostomia, and mucositis, were extracted from RT examination reports. Additionally, the total as well as the selective radiation dosage in the pharyngeal constrictor muscles were extracted from irradiation protocols and correlated with clinical variables. The cancer-specific survival (CSS) was determined in all patients and was used as the main oncological endpoint.

### 2.3. Dysphagia and Swallowing

We differentiated whether patients suffered from dysphagia before (pre-treatment dysphagia) or after the last irradiation setting (post-treatment dysphagia) and whether dysphagia improved or persisted over time (long-term or follow-up dysphagia). Evaluation and assessment of pre- and post-treatment dysphagia were performed retrospectively by evaluating patient records regarding symptomatic dysphagia. In contrast, long-term dysphagia was prospectively evaluated in patients with at least 5-year disease-free survival. Thus, appropriate patients (*n* = 26; 52%) were finally evaluated with either VFS or FEES after a mean follow-up of 74 ± 7 months (Figure 1). The PAS was applied as previously described to differentiate between normal swallowing (PAS 1), penetration (PAS 2–5), and aspiration (PAS 6–8) [5].

### 2.4. Statistical Methods

Statistical analyses were performed using SPSS version 27.0 software (IBM SPSS Inc., Armonk, NY, USA). Figures were created using GraphPad Prism version 9.0 software (GraphPad, San Diego, CA, USA). Unless otherwise specified, data are reported as mean ± standard error of the mean (SEM). Descriptive statistics were used for the analysis of demographic and clinical data. Chi-Square test and independent-students *t*-Test were applied to compare nominal variables and analyze the means of two normally distributed variables, respectively. Univariate binary logistic regression analysis was applied to evaluate the impact of different clinical variables on dysphagia throughout the observation period. Odds Ratios (ORs) and corresponding 95% confidence intervals (CIs) are indicated. Log Rank test was performed and Kaplan–Meier curves were illustrated for survival analyses. All tests were performed two-sided and *p*-values below 0.05 were considered statistically significant. No adjustments for multiple testing have been presented in the main tables as the study’s aims are rather exploratory than confirmatory. However, we performed *p*-value corrections via Bonferroni-Holm and added these values in table notes.

### 2.5. Ethics Approval

This study was approved by the ethics committee of the Medical University of Vienna (EK no. 1758/2017).

## 3. Results

### 3.1. Study Cohort

In total, 50 patients were evaluated including 12 females (24%) and 38 males (76%) with a median patient age of 73 ± 10.5 years (range 36 to 95 years). Regarding primary tumor site, SCCs were most commonly located at oral cavity (*n* = 23; 46%) followed by oropharynx (*n* = 16; 32%), hypopharynx (*n* = 8; 16%), and larynx (*n* = 3; 6%), respectively. We had 20 (40%) T1–T2 tumors and 30 (60%) T3–T4a tumors with positive neck nodes in 44 (88%) cases (Table 1). Among all patients, 10 (20%) had stage III and 40 (80%) had stage IV HNSCCs.

### 3.2. Therapy

RT was applied in all patients with a median total radiation dose of 64.51 ± 7.3 Gy at the tumor site. In comparison, those 34 patients (68%) with separate neck irradiation received a median dose of 52.2 ± 15.7 Gy, respectively. Importantly, the median radiation dose of the pharyngeal constrictor muscles was 61 ± 7.12 Gy (*n* = 45, the remaining 5 patients underwent irradiation at external centers). However, the majority of patients underwent surgery with adjuvant RT (*n* = 17; 34%) or CRT (*n* = 13; 26%). Primary CRT, in turn, was applied in 14 (28%) patients, while 6 (12%), particularly elder patients, solely received RT with curative intent. Further treatment details, irradiation amount, neck dissection, tracheostomy, or free flap use are descriptively summarized in Table 2.

### 3.3. Dysphagia

Of note, all patients suffered from dysphagia at any time during therapy or surveillance. Pre-treatment dysphagia was noticed in 24 patients (48%), whereas 46 patients (92%) complained about post-treatment dysphagia. Those four patients without dysphagic symptoms included laryngeal (*n* = 1), hypopharyngeal (*n* = 1) and oral cavity (*n* = 2) carcinomas. During the follow-up, post-treatment dysphagia rate dropped from 92% (*n* = 46) to 24% (*n* = 12), which was significantly different (*p* < 0.001). Otherwise, 38 patients (76%) achieved unaffected oral nutrition after curative RT. Neither age nor gender has significant impact on the occurrence of dysphagia (*p* = 0.584; *p* = 0.333; Table 2). Interestingly, there was a trend toward a higher risk for long-term dysphagia in patients with higher irradiation doses in the pharyngeal constrictor muscles (*p* = 0.175).

### 3.4. Risk Factors for Dysphagia

Next, we were interested in any risk factors that may contribute to the risk of pre-, post-, or long-term dysphagia. T3 and T4a SCCs indeed showed a 3.3-times higher risk for pre-treatment dysphagia (OR 3.3; *p* = 0.053). Similarly, patients with oropharyngeal or hypopharyngeal tumors also tended towards pre-treatment dysphagia (OR 2.92; *p* = 0.073). In sum, especially T3–T4a tumors originating from the oro—or hypopharynx carried the highest risk for pre-treatment dysphagia (OR 9.26; *p* = 0.009; Table 3).

Three months after the end of RT, patients with positive neck nodes had a 10-fold increased risk for post-treatment dysphagia (OR 10.53; *p* = 0.037). Four patients who suffered from T4a tumors mainly located in the oral cavity (3/4) became G-tube dependent during RT. In two of these, G-tube could be removed after satisfactory swallowing rehabilitation.

In turn, those patients who required free flap reconstruction showed the highest risk for long-term dysphagia at all (OR 6.10; *p* = 0.022), followed by T3–T4a OPSCC and HPXSCC (OR 4.42; *p* = 0.037; Table 3).

### 3.5. Long-Term Dysphagia and Penetration-Aspiration Scale

**Figure 2:**
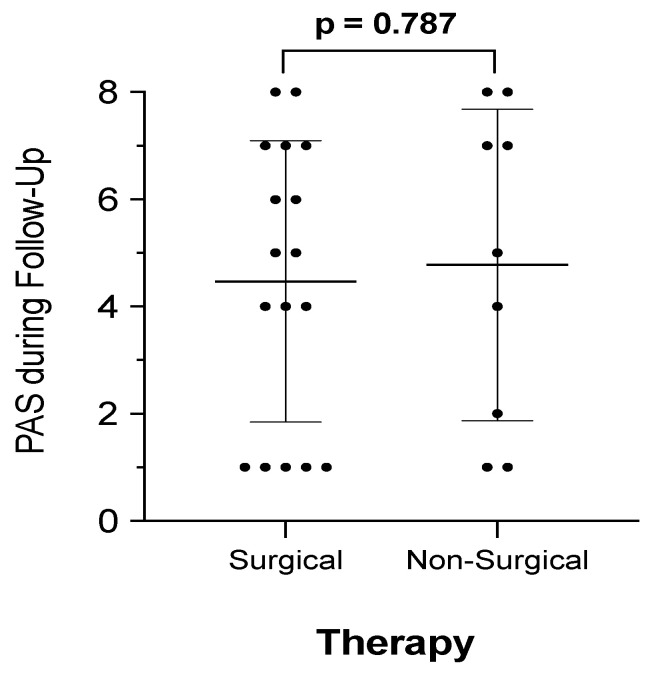
**PAS score and therapy**. Penetration-Aspiration-Scales (PAS) were available in 26 patients within follow-up. Patients were dichotomized into those who received surgical therapy, including surgery and radiotherapy (RT) or chemoradiotherapy (CRT), compared to patients who underwent non-surgical therapy, such as primary RT or CRT. Mean ± 95% Confidence-Intervals are indicated.

In addition to subjective assessment of pre- and post-treatment dysphagia, we performed VFS or FEES and applied the PAS to rate swallowing outcomes in 26 (52%) patients. The PAS score was significantly higher in patients with post-treatment dysphagic patients compared to non-dysphagic ones (*n* = 24; 4.9 vs. *n* = 2; 1.0; *p* < 0.001). During surveillance, the PAS score was 5.0 ± 2.5 in dysphagic patients (*n* = 8) compared to 4.4 ± 2.8 (*n* = 18) in non-dysphagic patients (*p* = 0.394). Moreover, PAS scores were not significantly affected by surgical procedures (*p* = 0.787), as illustrated in Figure 2. Data further demonstrate that swallowing malfunction and subsequently perception of dysphagia improves in 16 of 24 dysphagic patients (66.7%). However, a median PAS score ranging from 4.0 to 5.5 also indicates that the majority of long-term dysphagic patients showed signs of laryngeal penetration but the absence of aspiration. This was also proven by binary logistic regression analysis, demonstrating that patients with laryngeal penetration and incomplete clearing (PAS > 3) had a 4-times higher risk for long-term dysphagia (OR 4.42; *p* = 0.037).

### 3.6. Radiation Related Side-Effects

Finally, we evaluated the effect of common radiation-induced side effects on dysphagia development. Among these, mucositis represented the most common radiation-related side-effect, followed by erythema, xerostomia, dysgeusia, and soor in 36 (72%), 31 (62%), 28 (56%), 19 (38%), and 8 (16%) patients, respectively. Particularly xerostomia (OR 5.77; *p* = 0.019) and dysgeusia (OR 9.9; *p* = 0.036) significantly affected the subjective perception of dysphagia (Table 4). However, neither xerostomia nor dysgeusia significantly correlated with PAS score, age, gender, BMI, or radiation dose (data not shown).

### 3.7. Oncological Outcome

**Figure 3:**
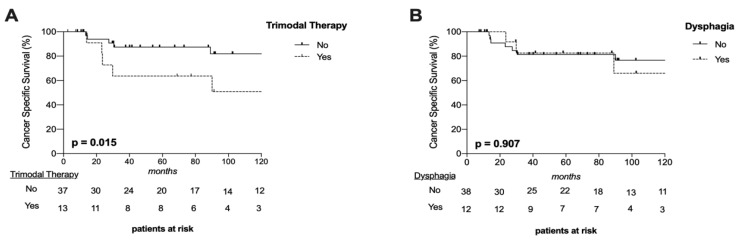
Survival Curves. Cancer-specific survival (CSS) was significantly worse in patients after trimodal therapy (surgery and chemoradiotherapy) (**A**) but not in long-term dysphagic patients (**B**).

In total, 15 patients (30%) deceased and most commonly from tumor-related causes resulting in a 1 y-, 3 y-, and 5 y-cancer-specific-survival (CSS) of 100%, 81.3%, and 81.3%, respectively. CSS was significantly affected by applied therapy (*p* = 0.015, Figure 3A) but not by tumor site (*p* = 0.734), T-classification (*p* = 0.489) or presence of dysphagia (*p* = 0.907, Figure 3B). Those 12 patients who required trimodal therapy (Surgery + CRT) due to additional risk factors, such as incomplete resection (*n* = 6), perineural invasion (*n* = 3), ECE (*n* = 2) or combination of ECE and incomplete tumor resection (*n* = 1), showed the worst CSS with a 5 y-CSS of 63.6%.

## 4. Discussion

Dysphagic symptoms with little complaints up to incapability of oral nutrition are common symptoms associated with advanced tumor disease or its treatment [15]. For obvious reasons, the oncological outcome must always be the decisive parameter for treatment choice. However, an increasing number of studies highlight the importance of the functional outcome and morbidity on the QoL of cancer patients [1,2,4,11]. RT is particularly associated with significant long-term effects related to associated tissue damage, such as fibrosis formation [16]. This highlights the importance of swallowing in high-stage HNSCC patients again. Nevertheless, correlation to long-term outcomes has been rarely addressed [10]. We thereby evaluated pre-treatment, post-treatment, and long-term follow-up dysphagia appearance in 50 patients with stage III-IV HNSCCs. All patients underwent RT, either solely or in combination with either chemotherapy, surgery, or both.

Dysphagia itself describes any sensation associated with impaired swallowing of food and liquids not necessarily linked to pain. Although individual patients’ perceptions of a swallowing disorder may be similar, the severity of dysphagia is distinguishable and should therefore be objectified [17]. A recent meta-analysis revealed that 75.4% of studies evaluated swallowing disorders subjectively, while only 30.2% presented an objective instrumental assessment of swallowing [10]. Our own data reflects the heterogeneity of subjective and objective dysphagic symptoms, showing that objective PAS scores did not necessarily differ among dysphagic and non-dysphagic patients with subjective symptoms. However, we observed that the majority of subjective symptoms following any kind of irradiation do not predict objective findings but that patients with laryngeal penetration (PAS ≥ 3) indeed carry a 4-fold higher risk for long-term dysphagia [18].

All of our patients had undergone RT and each of them had experienced dysphagia at any time during their therapy. Interestingly, we found significant differences in patients with pre-treatment, post-treatment, or follow-up dysphagia related to different causes and pathogenesis. Advanced stage (T3-T4a) oropharyngeal or hypopharyngeal SCCs showed the highest risk for pre-treatment dysphagia, which is most likely caused by tumor size or invasion. Conversely, almost all patients experienced post-treatment dysphagia, which was most likely related to RT-associated side effects like mucositis or xerostomia. Irradiation-related fibrosis of neural and vascular tissue with respective functional deficits may further impair post-therapeutic long-term swallowing function but barely plays a role in the acute setting [19,20]. There was also a trend toward worse swallowing outcomes and dysphagia in patients with higher irradiation doses in the constrictor pharyngeal muscles. Patients in our cohort were solely treated with former irradiation techniques, including more aggressive, less specific irradiation fields not sparing the constrictor pharyngeal muscles. As volumetric modulated arc therapy, an advanced form of intensity-modulated radiotherapy was introduced later in time, and the mean dose in the constrictor muscles has decreased since then [21]. This fact must be considered when interpreting our data if patients are informed about the side effects of adjuvant therapy and the likelihood of dysphagia occurrence, which may be even less these days. Xerostomia and dysgeusia represented the leading complaints associated with dysphagia that did not correlate with objective swallowing assessments. Thereby, those complaints do not pose suitable indicators for impaired swallowing process and objective examinations are strongly required if swallowing impairments are suspected. However, the subjective burden and the reduced quality of life caused by RT-induced xerostomia have been excessively reported before and represent a common sequel of RT that definitely needs stronger consideration [22,23].

It is also important to highlight that only four patients became G-tube dependent during RT and two of those achieved satisfactory swallowing recovery within follow-up. This indicates that RT-induced swallowing impairments are principally reversible. Therefore, a multidisciplinary team, including phoniatricians and Speech Language Therapists is necessary for the early identification of dysphagia and appropriate management [24]. Early-onset of functional swallowing therapy proved to be a statistically significant factor for successful swallowing rehabilitation [25]. Considering the modern radiotherapy techniques with more precise irradiation fields accompanied by less unintentional irradiation of surrounding tissue, RT itself with early swallowing rehabilitation seems to provide less risk for long-term swallowing malfunctions.

Patients undergoing free flap reconstruction carried the highest risk for long-term dysphagia, which has already been demonstrated [26]. Lahtinen et al. reported subjectively impaired swallowing in more than half of HNC patients two years after free flap reconstruction [27]. As poor nutritional status has shown to increase the risk of wound infections and poorer overall survival [15], the indication for G-tube should be made not too strict in patients at higher risk for post-treatment or long-term dysphagia to assure adequate nutrition during oncological therapy and rehabilitation. Although long-term dysphagia seems to be common in patients after CRT [20], only 15% of CRT-treated patients experienced dysphagia, and we did not observe any significant differences regarding dysphagia in the CRT cohort compared to those after surgery and PORT or RT alone.

Overall, our study has three weaknesses that partially weaken our results. First, we aimed to investigate the effect of different RT protocols on long-term swallowing outcomes in advanced staged HNSCC patients. Therefore, we applied strict inclusion and exclusion criteria resulting in a relatively small but homogenous patient cohort. Secondly, retrospective data collections always carry the risk of selection and information bias. Thirdly, evaluation of dysphagia is still challenging as there do not exist clear recommendations. Standardized swallowing evaluations and patient-reported outcome questionnaires are necessary. However, the strength of the study remains in the provision of subjective and objective swallowing data at different time points as well as prospective PAS evaluation. Altogether, our results further indicate that individual patients’ subjective swallowing disorder may not affect survival, although it indeed impairs QoL [1,28,29].

## 5. Conclusions

Almost all HNSCC patients receiving curative RT will develop swallowing malfunctions, but dysphagia significantly ceases over time. However, subjective perception of dysphagia does not necessarily correlate with objective criteria and underlines the necessity of objective assessments for swallowing disorders. Patients with certain risk factors for dysphagia, like oropharyngeal or hypopharyngeal tumor sites, as well as the necessity for free flap reconstruction, may benefit from earlier and more intensive speech-language and swallowing recovery.

## Figures and Tables

**Table 1:** Pre-Treatment and Follow-Up Dysphagia.

VARIABLES	Total	Pre-Treatment Dysphagia		Follow-Up Dysphagia	
	*n* (%)	*n* (%)	*p*-Value	*n* (%)	*p*-Value
**Sex**	50 (100)	24 (48)		12 (24)	
Female	12 (24)	4 (33)		3 (25)	
Male	38 (76)	20 (53)	0.333 ^a^	9 (24)	1.000 ^a^
**Age** (median ± SD)	73 ± 10.5				
**≤73 years**	27 (54)	14 (52)		5 (19)	
**>73 years**	23 (46)	10 (43)	0.584 ^a^	7 (30)	0.508 ^a^
**BMI** (mean ± SD)	25.2 ± 4.3				
<25	23 (46)	10 (43)		7 (30)	
≥25	27 (54)	14 (52)	0.567 ^a^	5 (19)	0.508 ^a^
**Tumor-Stage**					
Stage III	10 (20)	8 (80)		0 (0)	
Stage IV	40 (80)	14 (35)	**0.035** ^a^	12 (30)	0.092 ^a^
**T-Classification**					
T1–T2	20 (40)	6 (30)		2 (20)	
T3–T4a	30 (60)	18 (60)	0.080 ^a^	10 (33)	0.091 ^a^
**N-Classification**					
N0	6 (12)	3 (50)		1 (17)	
N1	9 (18)	7 (78)		1 (11)	
N2	33 (66)	13 (39)		10 (30)	
N3	2 (4)	0 (0)	0.655 ^a^	0 (0)	1.000 ^a^
**Tumor Site**					
Oral Cavity	23 (46)	7 (30)		6 (26)	
Oropharynx	16 (32)	9 (56)		4 (25)	
Hypopharynx	8 (16)	6 (75)		2 (25)	
Larynx	3 (6)	2 (67)	0.124 ^a^	0 (0)	0.798 ^a^

Clinical variables were assessed regarding pre-treatment dysphagia and dysphagia occurrence in follow-up. Absolute numbers (*n*) and corresponding percentages (%) are indicated within brackets. Significant results are presented in bold. Adjusted calculation for the *p*-value *p* = 0.035 is 0.245. ^a^ Chi-Square test.

**Table 2:** Detailed Clinical Treatment of the Observed Study group.

Therapy	Total	Follow-Up Dysphagia
	*n* (%)	*n* (%)
**Treatment**		
Surgery + PORT	17 (34)	6 (35)
CRT	14 (28)	4 (29)
RT	6 (12)	0 (0)
Surgery + CRT	13 (26)	2 (15)
**Radiation dose**		
Total irradiation	65.51 ± 7.3 Gy (*n* = 50, 100%)	-
Neck irradiation	52.2 ± 15.7 Gy (*n* = 34, 68%)	-
**Neck-Dissection**		
Yes	28 (56)	6 (21)
No	22 (44)	6 (27)
**Tracheostomy**		
Yes	17 (34)	6 (35)
No	33 (66)	6 (18)
**Free Flap**		
Yes	9 (18)	5 (56)
No	41 (82)	7 (17)

**Abbreviations:** PORT, post-operative radiotherapy; CRT, chemoradiotherapy; RT, radiotherapy.

**Table 3:** Binary Logistic Regression Analysis for Dysphagia.

CLINICAL VARIABLES	UNIVARIATE ANALYSIS								
	Pre-Treatment Dysphagia			Post-Treatment Dysphagia			Follow-Up Dysphagia		
	*OR*	*p*	*95% CI*	*OR*	*p*	*95% CI*	*OR*	*p*	*95% CI*
Sex (Female)	0.47	0.282	0.12–1.85	0.94	0.961	0.09–10.0	1.07	0.926	0.24–4.84
Age (≤73 y)	1.3	0.648	0.42–4.01	3.90	0.254	0.38–40.37	0.52	0.329	0.14–1.94
T3-T4a	3.30	0.053	0.99–11.1	0.47	0.531	0.05–4.90	4.50	0.073	0.87–23.3
N pos.	0.56	0.541	0.05–3.66	10.5	0.037	1.15–100	1.67	0.657	1.76–15.9
OPSCC + HPXSCC	2.92	0.073	0.91–9.43	*			1.38	0.632	0.37–5.05
T3-T4a AND OPSCC + HPXSCC	9.26	**0.009**	1.75–47.6	*			4.42	**0.037**	1.10–17.9
G-tube during irradiation (YES)	-	-	-	0.94	0.961	0.09–10.0	3.59	0.228	0.45–28.6
Free Flap (YES)	-	-	-	0.63	0.706	0.06–6.90	6.10	**0.022**	1.29–28.6
Tracheostomy (YES)	-	-	-	0.48	0.489	0.06–3.77	2.46	0.186	0.65–9.26
Neck-Dissection (YES)	-	-	-	1.30	0.801	0.17–10.0	0.73	0.632	0.20–2.67

**Note:** Significant *p*-values are presented in bold. Adjusted calculations for following *p*-values are presented in brackets *p* = 0.009 (0.054), *p* = 0.022 (0.198), *p* = 0.037 (0.269). **Abbreviations:** N pos., positive neck nodes; OR, odds ratio; G-tube, gastrostomy tube; y years; 95% CI, 95% confidence interval; * not calculable.

**Table 4:** Impact of radiation-associated side-effects and PAS score on follow-up dysphagia.

	Total	Follow-Up Dysphagia		
VARIABLES	*n* (%)	OR	*p*	95% CI
**Radiation Side-Effects**				
Soor	8 (16)	0.40	0.419	0.04–3.65
Dysgeusia	19 (38)	9.9	**0.036**	1.16–84.47
Erythema	31 (62)	1.30	0.767	0.33–5.10
Xerostomia	28 (56)	5.77	**0.019**	1.33–25.05
Mucositis	36 (72)	1.22	0.791	0.28–5.38
**PAS-Score**				
Retention	13 (26)	2.68	0.164	0.67–10.75
Penetration	13 (26)	4.42	**0.037**	1.10–17.86
Aspiration	12 (24)	1.88	0.389	0.45–7.87

**Note:** Significant *p*-values are presented in bold. Adjusted calculations for following *p*-values are presented in brackets *p* = 0.019 (0.152), *p* = 0.036 (0.252), *p* = 0.037 (0.222). **Abbreviations:** *n*, number of patients; OR, Odds Ratio; *p*, *p*-value; 95% CI, 95% confidence interval.

## Data Availability

Data is contained within the article and is available on request.

## Abbreviations

CRT	Chemoradiotherapy
CIs	Confidence intervals
CSS	Cancer-specific survival
ECE	Extracapsular extension
FEES	Fiberendoscopic (flexible) examination of swallowing
FU	Follow-up
HNC	Head and neck cancer
HNSCC	Head and neck squamous cell carcinoma
OPSCC	Oropharyngeal squamous cell carcinoma
HPXSCC	Hypopharynx squamous cell carcinoma
PAS	Penetration-Aspiration Scale
PORT	Post-operative radiotherapy
RT	Radiotherapy
SCC	Squamous cell carcinoma
VFS	Videofluoroscopy
